# The Influence of Pre-operative Pain and Anxiety on Acute Postoperative Pain in Cardiac Surgery Patients Undergoing Enhanced Recovery after Surgery

**DOI:** 10.4274/TJAR.2023.231477

**Published:** 2023-12-27

**Authors:** Aslıhan Aykut, Nevriye Salman, Zeliha Aslı Demir, Atakan Furkan Eser, Ayşegül Özgök, Serdar Günaydın

**Affiliations:** 1 University of Health Sciences Turkey, Ankara Bilkent City Hospital, Clinic of Anaesthesiology and Reanimation, Ankara, Turkey; 2 University of Health Sciences Turkey, Ankara Bilkent City Hospital, Clinic of Cardiovascular Surgery, Ankara, Turkey

**Keywords:** Cardiovascular and thoracic anaesthesia, enhanced recovery after surgery, multimodal analgesia, pain, preoperative anxiety

## Abstract

**Objective:**

Perioperative multimodal analgesia is an important step in enhanced recovery after surgery (ERAS) care. Many factors, such as preoperative chronic pain and anxiety, may provide information about the expected postoperative pain. In this study, we evaluated preoperative pain and anxiety and investigate their effects on acute postoperative pain in patients undergoing elective cardiac surgery.

**Methods:**

After ethics committee approval, 67 consenting patients undergoing on-pump cardiac surgery under the ERAS program were included in our prospective observational study. Pre- and postoperative pain scores were obtained using a numeric rating scale (NRS) at rest and during movement. Preoperative anxiety was assessed on a 0-10 scale, and data were recorded. The relationships between pre-operative pain/anxiety and postoperative pain were evaluated using correlation analysis.

**Results:**

In preoperative pain assessment, the percentage of patients with a pain score above 4 with NRS was 1.5%, regardless of whether they were at rest or mobilize. In postoperative pain assessment, there were 20.9% and 34.3% patients with NRS >4 at rest and mobilization, respectively. 7.5% of patients had preoperative anxiety of grade 5 or higher. While preoperative pain was not correlated with postoperative pain, preoperative anxiety had a moderate positive correlation with postoperative pain (r=0.382, *P*=0.003).

**Conclusion:**

The prevalence of preoperative pain in patients who underwent cardiac surgery is quite low and is not associated with postoperative pain. There is also a significant relationship between the severity of preoperative anxiety and postoperative pain.

**Main Points**

• Enhanced recovery after surgery (ERAS) recommends perioperative pain management with multimodal, non-opioid medications and detailed planning.

• Identification of preoperative pain predictors may enable patients at high risk of postoperative pain to receive personalized and successful treatment.

• The incidence of preoperative pain in cardiac surgery patients is low and not associated with postoperative pain. We believe that preoperative pain assessment does not contribute to pain management in cardiac surgery patients undergoing ERAS.

• We found that preoperative anxiety is associated with postoperative acute pain; therefore, we believe that interventions to prevent anxiety in the ERAS protocol will also contribute to postoperative pain management.

## Introduction

Postoperative pain is a cause of concern in patients undergoing heart surgery. Studies show that 47-75% of patients experience pain in the postoperative period, and it is often severe and undertreated. Patients recovering from cardiac surgery present a challenge when it concerns pain management because of the different characteristics of each patient and each procedure. Different methods of assessing postoperative pain in cardiac surgery patients have been validated.^1^

The enhanced recovery after surgery (ERAS) program uses multimodal and transdisciplinary approaches to reduce stress response and complications, eliminate postoperative pain, prevent the known side effects of opioids, and weaken the catabolic process. Providing perioperative multimodal analgesia constitutes an important step in these care pathways. Studies investigating the ERAS program, which is still an evolving intervention, have used paracetamol, gabapentin, non-steroidal anti-inflammatory drugs, and opioids in a multimodal analgesia regimen.^2^ Many factors such as preoperative pain, preoperative opioid use, previous postoperative pain experience, inappropriate patient expectations, surgical outcome anxiety, psychological factors, and functional pain can provide information about the expected postoperative pain in the patient evaluated preoperatively.^3,4^ By identifying these risks, postoperative pain management can be provided in a patient-specific manner. It has been stated that in noncardiac surgeries where ERAS is applied, patients may have some pain in the pre-operative period, and this should be evaluated in the pre-operative period.^5^ However, no study has evaluated the presence and severity of preoperative pain in terms of cardiac surgery. The effect of preoperative education on pain relief has mostly been investigated.^6^ It has been emphasized that preoperative anxiety levels are moderate and severe in patients undergoing cardiac surgery and that the presence of anxiety is associated with high postoperative pain scores.^4^

Because pain and anxiety are relatively subjective symptoms that show ethnic, identity, and national differences, we sought to determine the pre-operative pain and anxiety status in our own patient group, i.e., patients preparing for heart surgery, within the scope of the ERAS programs we currently implement at one of the largest cardiac surgery centers in our country. The aim of this study was to evaluate the presence of preoperative pain and anxiety in ERAS patients undergoing elective cardiac surgery and to investigate their effects on acute postoperative pain.

## Methods

This prospective, observational study was performed in conformance with the principles of the Declaration of Helsinki and was validated by the Ankara City Hospital No. 1 Clinical Research Ethics Committee (approval no: E1-22-2613, date: 15.06.2022). After written informed consent was obtained, 67 consecutive adult patients scheduled for elective open cardiac surgery in an ERAS program in 2023 were observed throughout the perioperative period. Adult patients who had undergone open cardiac surgery with cardiopulmonary bypass (CPB) within the scope of the ERAS protocol were included in the study. Patients with local anaesthetic allergy, body mass index greater than 35 kg m^-2^, emergency or re-do surgery, off-pump surgery, transplantation surgery, vascular surgery, age younger than 18 years, American Society of Anesthesiologists class IV (severe organ dysfunction), alcohol-drug use, and patients who died during or immediately after the operation were excluded from the study.

All patients received peroral pregabalin (150 mg) and antibiotic prophylaxis with cefazolin sodium (1000 mg) intravenously preoperatively. They were visited by a physiotherapist and started respiratory exercises 24 h before the operation. Patients had 6-8 h of fasting but drank 400 mL 12.5% maltodextrin 2 h before surgery. In the operating rooms, pulse oximetry, five-channel electrocardiography, and bispectral index monitoring (BIS™, Covidien, MN, ABD) were performed. 18 G and 16 G peripheral intravenous catheters and a radial arterial catheter were inserted under local anaesthesia.

Erector spinae plane (ESP) block was performed in the operating room during the pre-anaesthesia period with the patient in the prone position. A linear ultrasound transducer (PHILIPS Affiniti 50 color Doppler ultrasound device, Philips L12-5 50 mm linear array transducer) was placed in a longitudinal orientation 2.5-3 cm lateral to the T5-T6 spinous process. Three muscles were identified, and an 80-mm 21G block needle (Pajunk needle SonoPlex STIM 21x80 mm) was introduced in a cephalic-caudal position until its tip was inserted into the interfascial plane between the rhomboid major and erector spinae muscles. The injection was confirmed by observing a linear spread of the fluid (bilateral 20 mL 2.5%) at the targeted injection site. Preoperative single-shot bilateral ESP block was applied by AD and AO (Prof, MD), who have routinely applied ESP blocks in our clinic over the last three years.

Anaesthesia was administered intravenously with propofol (2-2.5 mg kg^-1^), fentanyl (2 mg kg^-1^), rocuronium (0.8 mg kg^-1^), and lidocaine (1 mg kg^-1^). General anaesthesia was maintained with inspiratory sevoflurane concentrations of 1.5-2.0%, titrated to achieve a BIS of 40-60, remifentanil infusion (0.05-0.25 mcg kg^-1^ min^-1^) and intermittant rocuronium. The following intubation, a protective ventilation strategy was used by applying 7 mL kg^-1^ tidal volume and 5-8 cm H_2_O positive end-expiratory pressure. A jugular central venous catheter was inserted under ultrasound guidance. Following harvesting and adequate activated clotting time (>480 s), arterial and venous cannulation were performed, and CPB was initiated. CPB was performed with moderate hypothermia (28-31 °C) and alpha stat strategy. Hemoglobin concentrations were maintained above 7.5 g dL^-1^ and glucose levels were maintained under 200 mg dL^-1^ during operation, and 100 mg lidocaine and 1.5 g magnesium were administered prior to cross-clamp removal according to our institutional approach.  At completion of CPB, heparin was replaced with protamine in a 1:1 ratio. Because of the short recovery time of sevoflurane and remifentanil, 0.5 mg kg^-1^ midazolam and 1 mg kg^-1^ tramadol were administered to the patients at the end of the operation. Paracetamol (1 g) was applied at sternal closing and repeated every 8 h. Following extubation, the severity of pain was assessed at rest and during movement using a 10-point numerical rating scale (NRS) for pain (0=no pain and 10=worst imaginable pain). Pain evaluation was performed based on all pain (sternum, saphenous and jugular regions, back, chest) in the 6th hour after extubation. In the setting of mild to severe postoperative pain (NRS for pain >4), a clinical bolus tramadol (1.5 mg kg^-1^) was administered to the patient as a rescue analgesic. In the postoperative intensive care unit, patients with complete orientation and cooperation, no significant haemodynamic problems, spontaneous breathing and PaO_2_ above 70 mmHg with 40% fractionated oxygen inhalation and no carbon dioxide retention were extubated. There was no duration of mechanical ventilation exceeding 8 h. The patients were extubated in 6-8 h and started oral intake 2 h following extubation. They were visited by a physiotherapist and dietician as soon as they were extubated to start respiratory exercises and to check if any additional nutritional support was needed. Major lines were removed 12 h after extubation, and the patients were transferred to the surgical wards. Demographic and intraoperative data were obtained. Preoperative anxiety was evaluated on a scale of 0-10 (NRS), and preoperative pain and anxiety were assessed on the day before surgery. Patient satisfaction in the postoperative period was also questioned on the first postoperative day, with 0 being “very dissatisfied” and 10 being “very satisfied”.

### Statistical Analysis

IBM SPSS.29.0 software was used for all data analysis. Descriptive statistics are presented as absolute numbers (n) and percentages (%) for categorical variables, the median‐interquartile range (25^th^-75^th^ percentiles) for non-normally distributed data, and the mean ± standard deviation for normally distributed data. The relationships between pre-operative pain/anxiety and postoperative pain were evaluated using Spearman’s rho correlation analysis.* P* < 0.05 was considered statistically significant. The sample size of our study was determined by the number of patients in the specified date range. After the statistical analysis was completed, a post-hoc power analysis was performed. In IBM SPSS program, β was calculated 0.92 when the correlation coefficient of preoperative anxiety and postoperative pain was r=0.382, α=0.05 and n = 67.

## Results

A total of 67 adult ERAS protocol patients who underwent elective cardiac surgery with CPB at our tertiary cardiac center were included from March 2023 to August 2023, and all patients were analyzed. The mean age of patients was 62.1 years, male gender was 80.6%, and body mass index was 28.45. Coronary artery bypass surgery was performed in 61% of the patients. The most frequent diseases were hypertension and diabetes mellitus (43%, 28%) (Table 1).

The rate of patients with a preoperative pain level NRS>4 was 1.5% and 1.5% at rest and with movement, respectively. In the postoperative period, pain >4 assessed by NRS was observed in 20.9% of patients at rest and 34.3% with movement and rescue analgesics were administered to these patients (Table 2).

While no anxiety was detected in 65.6% of the patients, 34.3% had preoperative anxiety of grade 1 or above. The rate of patients with an anxiety level of NRS 5 was 7.5% (Table 2). In the patient satisfaction survey, 91.1% of patients were satisfied with the care provided (Table 2).

While there was no correlation between preoperative pain and postoperative pain in the patient by Spearman’s rho test, it was determined that preoperative anxiety had a moderate correlation with postoperative pain (*P*=0.003), and as the severity of anxiety increased, the severity of postoperative pain also increased (Table 3).

## Discussion

The ERAS protocol recommends pain management with detailed planning, perioperatively, multimodally, and often with non-opioid medications. It is mentioned that preoperative pain is often ignored in ERAS programs. In this study, we evaluated the frequency of preoperative pain in our cardiac surgery patients and found that the rate of patients with NRS ≥4 was only 1.5%. We also did not find any correlation between pre- and postoperative pain.

Questioning a patient’s current preoperative pain levels can help optimize postoperative pain management. The patient’s psychological state and dissatisfaction with previous hospital experiences are associated with a high risk of postoperative pain.^7^ In addition, learning about a patient’s initial pain and considering how to manage it preoperatively can help identify potential barriers. If there was a negative experience with pain management after a previous surgery, if the pain was not adequately resolved with a non-opioid pain prescription, or if there was chronic opioid use in the pre-operative period, these patients can be expected to experience more pain in the postoperative period. One study showed that the preoperatively operated knee had a greater response to suprathreshold heat stimuli than the other. Therefore, one of the causes of hyperalgesia in the affected knee is peripheral nerve sensitization caused by inflammation.^8^ Before cancer surgery, the patient may experience pain depending on the location of the cancer, or the sadness caused by the cancer diagnosis may lower pain thresholds, which may also cause preoperative pain, similar to that in some orthopedic surgeries. However, it seems that preoperative pain is not a major issue demanding attention for cardiac surgery patients. In our clinic, ERAS application is used to provide information about the operation process, preoperative oral pregabalin is administered, and preoperative pain and anxiety assessments are performed. Accordingly, we believe that because very low and clinically insignificant preoperative pain is observed in the ERAS programs of cardiac patients who are already walking with great devotion, there is no need to make extra effort to detect this. Although preoperative chest pain may be more common in emergency cases, it is rare in elective cases.

In our results, a positive relationship was found between the severity of preoperative anxiety and postoperative pain. Although we aimed to reduce anxiety in our ERAS patients with pregabalin medication and information, 34.3% of the patients had anxiety NRS ≥1, and 7.5% of the patients had NRS ≥5. Although it is expected that a patient scheduled for heart surgery will be anxious considering the importance and magnitude of the surgery, it is clinically noteworthy that the severity of anxiety increases postoperative pain. In this case, it seems necessary to make more diverse interventions to relieve preoperative anxiety. The effort spent for this purpose is worth making as it can also contribute to postoperative pain relief. Studies have shown that preoperatively anxious patients have higher postoperative pain.^9,10,11^ High levels of preoperative anxiety can lead to intraoperative hemodynamic problems and increased need for analgesics.^11^ The European Society of Anesthesiology guidelines recommend that anxiety assessment be included in pre-operative assessments.^12^ One of the most validated and widely used instruments to evaluate preoperative anxiety in cardiac surgery is the state-trait anxiety inventory (STAI).^13^ However, answering 40 state-reporting questions is time-consuming for the patient and doctor and may not always be done properly. In our study, instead of performing a detailed STAI test, we used the NRS scale, which patients can easily understand and will not take up much of the doctor’s time. Even with this simple NRS scale setup, we could determine that preoperative anxiety was correlated with postoperative pain. Accordingly, in such a busy workload, simple NRS scoring could work quite well.

## Conclusion

In this study, postoperative pain was assessed once at the time when it was expected to be most severe. However, monitoring and treatment procedures to provide analgesia were continued in clinical management. Advanced techniques, such as heat or electrical application, were not used to detect  pain in the pre-operative period. Detailed psychic tests were not performed to evaluate anxiety. ERAS applications are already carried out with the dedication of many people, so this study aims to make evaluations with easy and practical methods.

Postoperative pain, which is an important prognostic marker after cardiac surgery, is a multifactorial and complex phenomenon. In our study, we found that preoperative pain does not seem to be a major problem in cardiac surgery patients and has no effect on postoperative pain. Therefore, we believe that the addition of multimodal pain management to the ERAS protocol may cause extra labor loss. However, we found that preoperative anxiety was associated with postoperative pain. We believe that preoperative regulation of a modifiable factor such as anxiety and implementation of personal interventions to reduce stress may improve outcomes.

## Figures and Tables

**Table 1:**
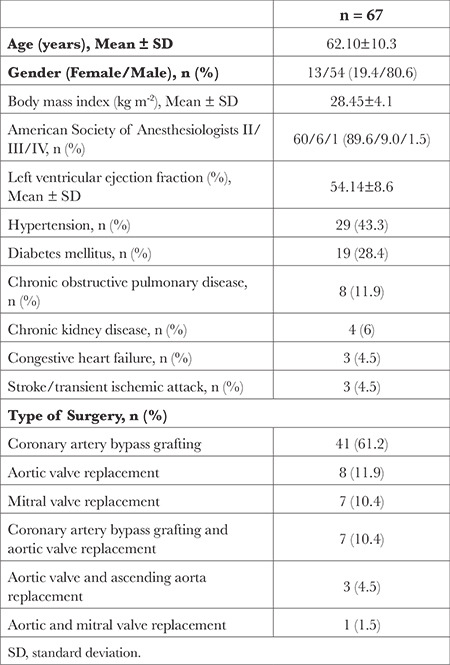
Demographic Data and Type of Surgery

**Table 2:**
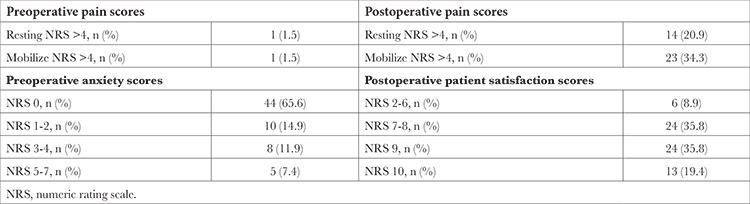
Preoperative and Postoperative Pain, Anxiety and Patient Satisfaction

**Table 3:**
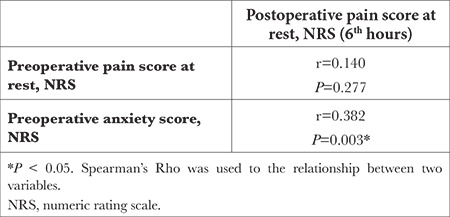
Correlation of Preoperative Pain and Anxiety with Postoperative Pain
